# Two new species of *Episymploce* Bey-Bienko, 1950 (Blattodea, Ectobiidae, Blattellinae) from China

**DOI:** 10.3897/zookeys.954.49738

**Published:** 2020-07-29

**Authors:** Ting-Ting Li, De-Xing Liu, De-Yi Qiu, Qiao-Yun Yue

**Affiliations:** 1 Zhongshan Customs Technology Center, Zhongshan 528400, Guangdong, China Zhongshan Customs Technology Center Zhongshan China

**Keywords:** Blattaria, cockroaches, Dictyoptera, identification key, taxonomy

## Abstract

Two new species of *Episymploce* Bey-Bienko from China are described. Nine individuals of *E. sichuanensis***sp. nov**. were collected from Sichuan Province and four individuals of *E. maxima*, **sp. nov**. were collected from Guangxi Province. Morphology, especially the wings, specialized abdominal tergum and genitalia of adults, are described and illustrated in detail. *Episymploce sichuanensis***sp. nov.** is similar to *E. kunmingi* (Bey-Bienko, 1969), but can be easily distinguished by the reduced wings, bifurcated two processes at the hind margin of the supra-anal plate, and the unspecialized first abdominal tergum (T1). *Episymploce maxima***sp. nov.** is similar to *E. taiheizana* Asahina, 1979 but is distinguished by its large size, the lateromedial margins of the subgenital plate without processes, and the unspecialized T1. A key to the recorded *Episymploce* species from China is provided in this paper.

## Introduction

The genus *Episymploce* was established by Bey-Bienko in 1950, with the type species *E. paradoxura* Bey-Bienko, 1950, who later described three other new species, *E. marginata* Bey-Bienko, 1957, *E. popovi* Bey-Bienko, 1957, and *E. uncinata* Bey-Bienko, 1969. Princis (1969, 1971) recorded six species of *Episymploce*, five of which originated from China. In 1979, Asahina (1979) redescribed the Japanese species *E. amamiensis* Asahina, 1977, reinterpreted the genus and described another two new species, *E. princisi* Asahina, 1979 and *E. taiheizana* Asahina, 1979. Asahina (1979) considered *Ischnoptera multiramosa* Karny, 1915, recorded by Karny and Shiraki, incompletely documented, while Princis (1969) pointed out a nomenclatural error and changed its name to *E. karnyi* Princis, 1969, but no detailed description was provided. Asahina (1979) renamed *E. karnyi* Princis, 1969 as *E. princisi* Asahina, 1979 and redescribed it. Roth (1985) then renamed *E. princisi* Asahina, 1979 to *E. asahinai* Roth, 1985 and redescribed the species again. Liu et al. (2017) also considered *E. karnyi* Princis, 1969 and *I. multiramosa* Karny, 1915 were synonyms of *E. asahinai* Roth, 1985. *Phyllodromia formosana* Shiraki, 1908 and *I. yoshinoe* Shiraki, 1931 from Taiwan were identified as subspecies of *E. formosana* (Shiraki, 1907) by Asahina (1979). Roth (1987d), Wang (2006) and Liu et al. (2017) considered *E. formosana formosana* (Shiraki, 1907) was a synonym of *E. formosana* (Shiraki, 1907).

Roth (1986) supplemented the genus and reclassified five species of *Symploce* as *Episymploce*. He considered that *E. taiwanica* (Bey-Bienko, 1969) was a synonym of *E. sundaica* (Hebard, 1929). Wang (2006) and Liu et al. (2017) agreed with Roth. Roth (1986) transferred six species of *Symploce* to *Episymploce*, and considered that *E. castanea* (Hanitsch, 1933) was a synonym of *E. ussuensis* Roth, 1985. Roth (1987a, 1987b, 1987c, 1987d) described 41 species of *Episymploce* from six countries, some being new records, of which 27 species and two subspecies were distributed in China, and a key to these Chinese species was provided.

Guo and Feng (1985) established *Asymploce* Guo & Feng, 1985, and recorded two new species, *Asymploce rubroverticis* Guo & Feng, 1985 and *A. hunanensis* Guo & Feng, 1985 from China, but Roth (1991) subsequently revised this genus, placing it as a synonym of *Episymploce*, and placed these two species into *Episymploce*. Roth (1997) transferred 16 species of *Symploce* to *Episymploce* based on the supra-anal plate. In 2003, Roth (2003) transferred *S. guizhouensis* Feng & Woo, 1988 and *S. mamillatus* Feng & Woo, 1988 from *Symploce* to *Episymploce*. Wang at al. (2005) described a new species *E. daozhenana* Wang & Feng, 2005 from Guizhou of China.

By now, there are more than 70 species of *Episymploce* recorded globally, of which 39 species are recorded in China (Beccaloni 2014); a key of the published 39 species and the two new species reported here is provided in this paper.

## Materials and methods

On 6 April and 3 May 2014, the second author and another colleague collected specimens in Daheishan, Panzhihua County, Sichuan Province, and Nonggang Village, Longzhou County, Chongzuo City, Guangxi Province. The specimens were brought back to the laboratory for freezing, flattening of wings and limbs with parchment paper, pinning with needles, and drying for preservation. The tergum behind the seventh abdominal tergum (T7) of the male specimen was cut off, placed into a 1.5 ml centrifuge tube with 10% NaOH and digested at 70 °C for 30–45 min. After the digestion, NaOH was removed from the centrifuge tube, and the specimen was rinsed thrice with water before examination. The specimens were dissected and observed under a ZEISS Discovery V12 stereo microscope. Photographs were taken with a ZEISS/Smart Zoom5 and Canon EOS 5D Mark III, and illustrated with Adobe Photoshop CC 2017 software. After illustration, the genitalia were stored in 0.5 ml centrifuge tubes containing 50% glycerol. The type specimens were deposited in Zhongshan Customs Technology Center.

The terminology used in this paper follows Roth (1977, 1979, 2003).

## Taxonomy

### Episymploce

Taxon classificationAnimaliaBlattodeaEctobiidae

Bey-Bienko, 1950: 157.

2360E10D-5A15-5C3D-A3EA-A25E0B794DD6

#### Type species.

*Episymploce paradoxura* Bey-Bienko, 1950: 157.

#### Diagnosis.

According to the traits proposed by Bey-Bienko (1950), Asahina (1979) and Roth (1986), this genus can be described as follows: the tegmina and wings are fully developed. Wings cubitus anterior vein has 1–5 complete and 1–6 incomplete branches, and the triangular apical area is small, reduced or absent. The first abdominal tergum can be specialized or unspecialized; the seventh abdominal tergum is always specialized; right and left lateral plates of the ninth abdominal tergum are similar, or the size and shape are obviously different, and the apex can be with or without small spines. The supra-anal plate is asymmetrical, symmetrical, or approximately symmetrical, the apex of the posterior margin is invaginated, or slightly concave; the subgenital plate is asymmetrical. The anteroventral margin of the front femora is of Type A3, rarely Type B, or between Type A and Type B. The male left aedeagus is in the shape of a hook.

#### Distribution.

China; Indonesia (Sumatra, Sulawesi, Java, Flores); Japan; India; Laos; Vietnam; Philippines; Thailand; Borneo Island; Nepal; Burma; Malaysia; Singapore; Australia; Papua New Guinea.

**Remarks**. We agree with Roth (1987d), Wang (2006) and Liu et al. (2017) that *E. taiwanica* (Bey-Bienko, 1969) is a synonym of *E. sundaica* (Hebard, 1929), and agree with Roth (1987d) and Liu et al. (2017) that *E. karnyi* Princis, 1969 is a synonym of *E. asahinai* Roth, 1985. We also agree with Asahina (1979), Roth (1987d), Wang (2006) and Liu et al. (2017) that *E. formosana formosana* (Shiraki, 1907) is a synonym of *E. formosana* (Shiraki, 1907). So, *E. taiwanica* (Bey-Bienko, 1969), *E. formosana formosana* (Shiraki, 1907) and *E. karnyi* Princis, 1969 were not be included in the key below. Forty-one species in *Episymploce*, including all published 36 species, three subspecies and two newly described species are included in this key, which is adapted from Roth (1987d).

### Key to species of *Episymploce* from China (males)

**Table:** 

1	Anteroventral margin of front femur Type A3, rarely intermediate between Type A and B	**2**
–	Anteroventral margin of front femur Type B3	**8**
2	Supra-anal plate symmetrical	**3**
–	Supra-anal plate weakly asymmetrical or asymmetrical	**5**
3	Hind margin of supra-anal plate shallowly concave in middle and without papilla mesad	**4**
–	Hind margin of supra-anal plate shallowly concave on the apex and with a minute papilla mesad	***E. asahinai* Roth, 1985**
4	Subgenital plate asymmetrical, styles simple, left and right lateromedial margins with spine-like processes. Left and right lateral plateral of T9 almost same length, ventral margins of both plates with 3 spines near apex	***E. taiheizana* Asahina, 1979**
–	Subgenital plate asymmetrical, styles simple, left and right lateromedial margins without processes. Left and right plate plateral of T9 similar, ventral margins of both plates without spines	***E. maxima* sp. nov.**
5	Supra-anal plate divided	**6**
–	Supra-anal plate undivided, ligulate	***E. ligulata* Bey-Bienko, 1957**
6	Hind margin of subgenital plate with a U-or V-shaped excavation	**7**
–	Hind margin of subgenital plate without a U-or V-shaped excavation	**11**
7	Left lobe of supra-anal plate wider than right lobe, inner margin of supra-anal plate with a curved incision, inner margin apex with a small papilla	***E. mamillatus* (Feng & Woo, 1988)**
–	Left lobe of supra-anal plate wider than right lobe, inner margin apex of supra-anal plate without papilla	**8**
8	Left and right lateral plateral of T9 almost same length, posteroventral margins without spines	***E. sundaica* (Hebard, 1929)**
–	Left and right lateral plateral of T9 not similar, posteroventral margins with spines	**9**
9	Basolateral of subgenital plate without spine-like process, right inner ventral margins with a strong spine	***E. cheni* (Bey-Bienko, 1957)**
–	Basolateral of subgenital plate each with a spine-like processes	**10**
10	Left thickened margin of subgenital plate produced transversely truncated, right style long and straight covered with dense hairs	***E. subvicina* (Bey-Bienko, 1969)**
–	Left thickened margin of subgenital plate produced cylindrical, right style long and straight not covered with dense hairs	***E. vicina* (Bey-Bienko, 1954)**
11	Left and right processes crossed of supra-anal plate hind margin	**12**
–	Left and right processes uncrossed of supra-anal plate hind margin	**14**
12	Left and right ventral margins of T9 without serrations	**13**
–	Left ventral margins with tines, right ventral margins with or without serrations	***E. Princisi* (Bey-Bienko, 1969)**
13	Left and right lateral plates of T9 with spines on ventral margin	***E. malaisei* (Princis, 1950)**
–	Left lateral plate of T9 without spines on posteroventral margin, right lateral plate with or without spines	**15**
14	Ventral margins of T9 with a long spine. Hind margin apex of subgenital plate with a digitiform process	***E. malaisei externa* (Bey-Bienko, 1969)**
–	Ventral margins of T9 without spine. Hind margin apex of subgenital plate without process	***E. malaisei malaisei* (Princis, 1950)**
15	Left and right lobes of supra-anal plate with equal width, right apex spine-like, left one round	***E. dimorpha* (Bey-Bienko, 1958)**
–	Left and right lobes of supra-anal plate not equal width	**16**
16	Left and right processes of supra-anal plate joined	**20**
–	Left and right processes of supra-anal plate separate	**17**
17	Hind margin of both lateral plates of T9 transversely truncated, ventral margins projecting posteriorly with spine-like processes or without processes	**18**
–	Hind margin of left lateral plate of T9 obliquely truncated, posteroventral angles of right plate with slight processes	***E. quarta* (Bey-Bienko, 1969)**
18	Left thickened hind margin of subgenital plate spicular, right margin with an upright or curved hooklike style	**19**
–	Left thickened hind margin of subgenital plate strongly spinulose, right margin without upright style	***E. secunda* (Bey-Bienko, 1957)**
19	Right margin of subgenital plate with a large and upright style	***E. prima* (Bey-Bienko, 1957)**
–	Right margin of subgenital plate with a large and curved hooklike style	***E. tertia* (Bey-Bienko, 1957)**
20	Left and right processes of supra-anal plate curved to the same side of ventral margin	**23**
–	Left and right processes of supra-anal plate curved to different sides of ventral margin	**21**
21	Middle with fleshy elevation of T7 with a pair of fossae on each side	**22**
–	Middle without fleshy elevation of T7 with depressions on each side	***E. unicolor* (Bey-Bienko, 1958)**
22	Hind margin of supra-anal plate process on the middle part, chelate. Pronotum with black brown blotch	***E. tridens* (Bey-Bienko, 1957)**
–	Hind margin of supra-anal plate crevice on the middle part, apex of both lobe with curved long spine-like processes, directed along hind margin. Pronotum front margin and disk black brown, lateral and hind margin yellowish-brown	***E. hunanensis* (Guo & Feng, 1985)**
23	Right plate of subgenital plate with an irregular lamellar formation	***E. zagulajevi* (Bey-Bienko, 1969)**
–	Right plate of subgenital plate without an irregular lamellar formation	**24**
24	Left and right ventral margins of T9 apex with small spines	**25**
–	Left and right ventral margins of T9 with long spine-like processes	**27**
25	Hind margin of apex of supra-anal plate with two processes without bifurcate spine	***E. kryzhanovskii* (Bey-Bienko, 1957)**
–	Hind margin of apex of supra-anal plate with two processes with bifurcate spine	**26**
26	Hind margin apex of supra-anal plate with two bifurcate processes	***E. sichuanensis* sp. nov.**
–	Hind margin of supra-anal plate with right process bifurcate, left process spine-like	***E. kunmingi* (Bey-Bienko, 1969)**
27	Left and right lateral plates of T7 with a small fossea. Right lobe of apex of supra-anal plate spine-like, left lobe broadly with an adpressed transverse spine ventrally near apex	***E. spinosa* (Bey-Bienko, 1969)**
–	Left and right lateral plates of T7 with a depression. Left and right lobes of supra-anal plate spine-like, left lobe with a long spine ventrally near apex	***E. longiloba* (Bey-Bienko, 1969)**
28	Supra-anal plate divided in two	**31**
–	Supra-anal plate not divided	**29**
29	Middle of T7 with two fossea covered in hairs	***E. marginata* Bey-Bienko, 1957**
–	Middle of T7 with a pair broad fossea without hair covering	**30**
30	Supra-anal plate semitubular, left margin apex with a long style, right margin near apex with a long style, basolateral with a process	***E. popovi* Bey-Bienko, 1957**
–	Supra-anal plate weakly asymmetrical, triangular, left margin apex with two long styles, left and right basolateral without processes	***E. forficula* (Bey-Bienko, 1957)**
31	Hind margin of subgenital plate with V-shaped excavation	**32**
–	Hind margin of subgenital plate without V-shaped excavation	**36**
32	Right lobe ventrally of supra-anal plate with an adpressed transverse spine	***E. daozhenana* Wang & Feng, 2005**
–	Right and left lobe of supra-anal plate without spines	**33**
33	Left and right lateral plates of T7 with a small fossea	**34**
–	Left and right lateral plates of T7 without fossea	**35**
34	Left and right lateral plates basolateral of subgenital plate without processes, left plate terminating with a small spine, two styles long spine-like	***E. paradoxura* Bey-Bienko, 1950**
–	Left and right lateral plates basolateral of subgenital plate with processes, left plate process is 2.5 times longer than the right plate process, left style curved hooks directed across left side of plate, right style straight spine-like obliquely directed across supra-anal plate	***E. potanini* (Bey-Bienko, 1950)**
35	Left and right lateral plates basolateral of subgenital plate with process, left plate thickened, terminating with a small spine, styles dissimilar, their bases widely separated	***E. hassenzana* Roth, 1987**
–	Left and right lateral plates basolateral of subgenital plate with processes, left plate thickened, terminating without a small spine, both styles nearly touching basally, left style long spine-like, right style directed across right rear	***E. paravicina* (Bey-Bienko, 1969)**
36	Left and right ventral margins of apex of T9 with spines	**37**
–	Left and right ventral margins of T9 directed to long spine-like processes	**39**
37	Hind margin near left corner of supra-anal plate with a larger deflexed spine-like process, left margin apex thickened, hind margin medially with a pair of minute filamentous processes	***E. splendens* (Bey-Bienko, 1957)**
–	Hind margin of supra-anal plate each terminating with a spine-like deflexed process, left margin apex not thickened, hind margin medially without filamentous processes	**38**
38	Right and left lateral plates of T9 similar, hind margin transversely truncated, each with ventral margin terminating in a small spine	***E. formosana* (Shiraki, 1907)**
–	Left ventral margin of T9 with a small spine, right ventral margin without spine	***E. formosana yoshinoe* (Shiraki, 1931)**
39	Upper base of both sides of supra-anal plate black brown. A red inverted pentagram marking is formed on vertex, ocular and antennal areas	***E. rubroverticis* (Guo & Feng, 1985)**
–	Upper base of both sides of supra-anal plate not black brown. No red inverted pentagram marking present on the face	**40**
40	Left lateral plate of T9 with a narrow, apex spine-like, right plate short with a long, curved spine, not inserted in genital cavity. Pronotum with a pair of rust-chestnut spots	***E. uncinata* Bey-Bienko, 1969**
–	Left lateral plate of T9 with a short spine-like, right plate with a long spine, inserted in genital cavity. Pronotum yellowish-brown	***E. guizhouensis* (Feng & Woo, 1988)**

### Episymploce sichuanensis sp. nov.

Taxon classificationAnimaliaBlattodeaEctobiidae

6A3D8989-99A1-5AED-8B7F-22F7B0FDA0F2

http://zoobank.org/290ECF9F-4DA0-4EEF-9086-4AB258357B65

Figures 1–4 , 5–14

#### Specimens examined.

***Holotype***: 1 male, 26°38.48'N, 101°41.62'E, Daheishan, Panzhihua City, Sichuan Province, 6 April 2 014, coll. Ke-Liang Wu et De-Xing Liu. ***Allotype***: 1 female, ***paratype***: 7 males, allotype and paratype were collected together with holotype.

#### Diagnosis.

This species is similar to *E. kunmingi* (Bey-Bienko, 1969), but can be distinguished as follows: 1) tegmina and wings reduced, only reaching the second abdominal tergum (T2), while in *E. kunmingi* (Bey-Bienko, 1969) tegmina and wings reach the apex of abdomen; 2) T1 is unspecialized, whereas T1 is specialized in *E. kunmingi* (Bey-Bienko, 1969); 3) two inwardly curved bifurcate processes on the hind margin of the supra-anal plate, while in *E. kunmingi* (Bey-Bienko, 1969) right process bifurcate and left process spine-like.

**Figures 1–4.**
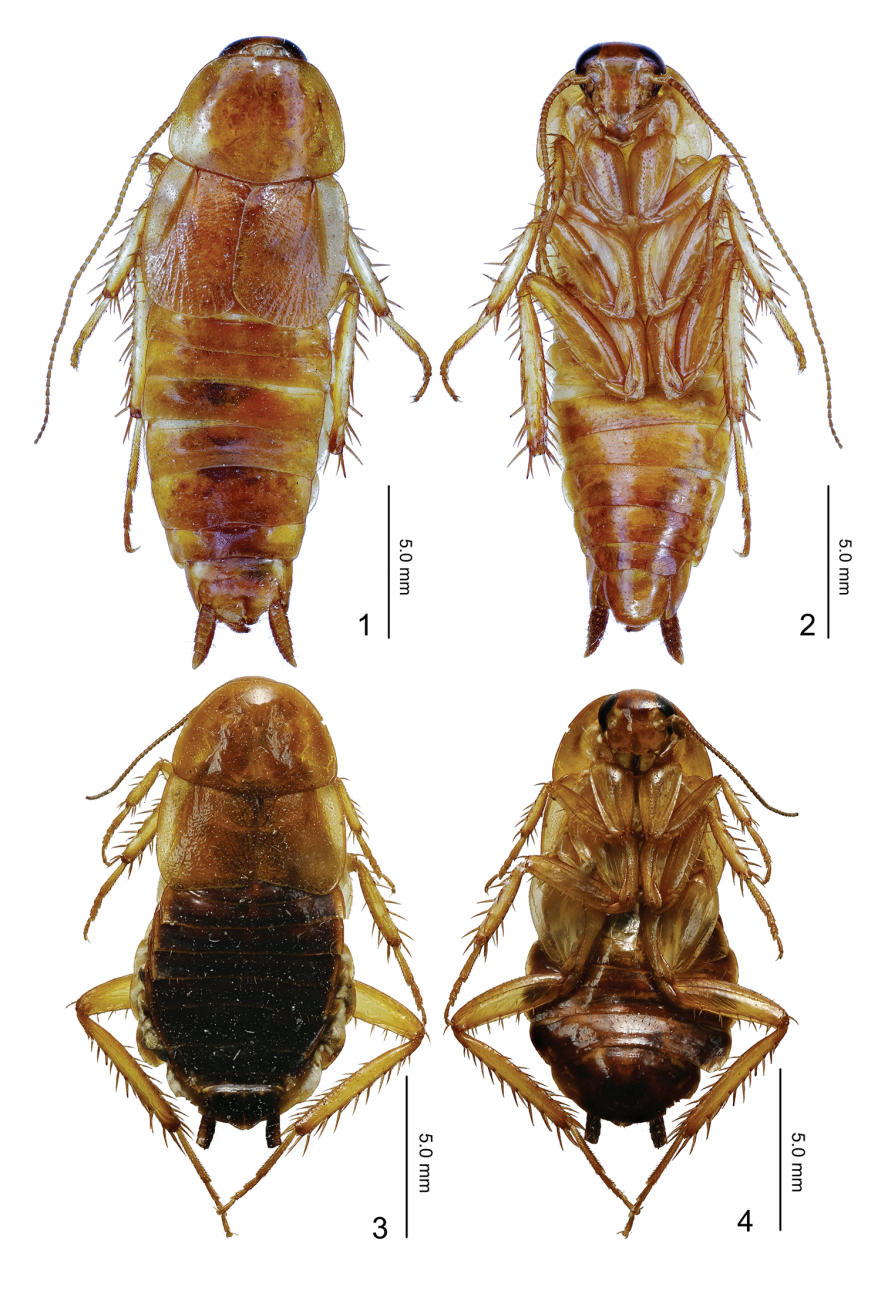
*Episymploce sichuanensis* sp. nov. **1** male, dorsal view **2** male, ventral view **3** female, dorsal view **4** female, ventral view.

#### Description.

Male, pronotum: length × width: 2.8–3.0 × 3.8–4.4 mm; tegmen: 3.5–4.0 mm; overall length (including tegmen): 16.2–17.9 mm. Female, pronotum: length × width: 3.2 × 5.0 mm; tegmen: 3.8 mm; overall length (including tegmen): 14.4 mm.

Small size. Body yellowish orange, head extending somewhat beyond pronotum, ocellus white, interocular space almost equivalent to ocellus space. Pronotum approximate ladder-like, hind margin wide. Tegmina and wings reduced, veins inconspicuous, reaching of T2. Anteroventral margin of front femur Type A3; the first tarsus of the hind leg longer than the sum of the remaining tarsi; tarsal claws symmetrical and unspecialized, arolium and pulvillus present. The T1 unspecialized; T7 specialized with a pair of approximately triangular depressions (Fig. 7); T9 asymmetrical with left side longer than right, apex margin with some small spines (Fig. 9). Male supra-anal plate asymmetrical, hind margin of lamina bilobed, left hind margin has two inwardly curved processes, apex process of which is bifurcate (Figs 6, 8); left side paraproct with three processes and right side with single process. Subgenital plate asymmetrical, basolateral with two processes, left process longer, and apex of right process curved; left of hind margin apparently thicker and covered strongly spinulose, middle hind margin with two spine-like processes reversed and outwardly with long styles (Fig. 10); left aedeagus hook-shaped (Fig. 11). Female is similar to male; abdominal tergum suffused with dark brown, supra-anal plate symmetrical, approximately triangular, apex concave (Fig. 14). Subgenital plate simple and hind margin rounded (Fig. 15).

#### Etymology.

Species name *sichuanensis* refers to the type locality.

#### Distribution.

China (Sichuan).

**Figures 5–15.**
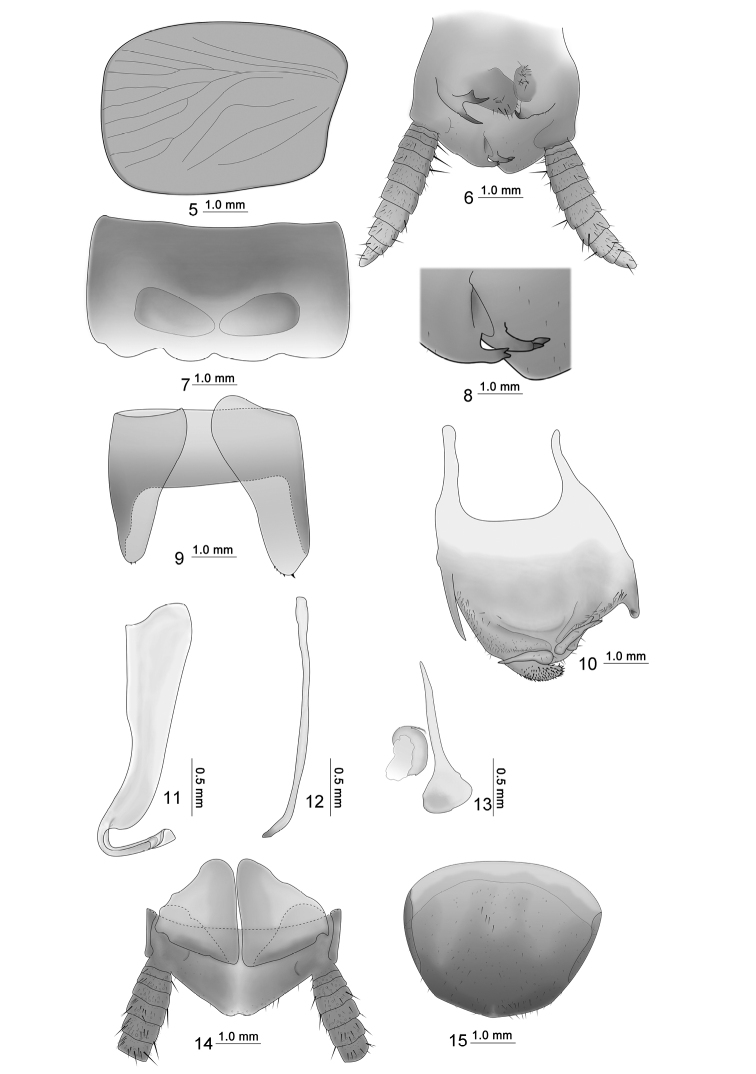
*Episymploce sichuanensis* sp. nov. **5** tegmen **6** male, supra-anal plate and paraprocts, ventral view **7** male, T7, dorsal view **8** male, bifurcate process of the supra-anal plate **9** male, T9, ventral view **10** male, subgenital plate, dorsal view **11** male, left aedeagus **12** male, median aedeagus **13** male, right aedeagus **14** female, supra-anal plate and paraprocts, ventral view **15** female, subgenital plate, ventral view.

### Episymploce maxima sp. nov.

Taxon classificationAnimaliaBlattodeaEctobiidae

F0883A5C-3FD2-576D-8069-ACCD62D041F9

http://zoobank.org/4C780B62-095A-48CD-9765-C7BD7EEAA95C

Figures 16–19 , 20–30

#### Specimens examined.

***Holotype***: 1 male, 22°28.26'N, 106°57.43'E, Nonggang Village, Longzhou County, Chongzuo City, Guangxi Province, 3 May 2014, coll. Ke-Liang Wu et De-Xing Liu. ***Allotype***: 1 female, ***paratype***: 1 male, 1 female, all speciemens were collected at the same place at the same time.

#### Diagnosis.

This species is similar to *E. taiheizana* Asahina, 1979, but can be distinguished as follows: 1) lateromedial margins of subgenital plate without processes, while with processes in *E. taiheizana* Asahina, 1979; 2) T1 was unspecialized, but T1 was specialized in *E. taiheizana* Asahina, 1979; 3) ventral margins of T9 without spines, but with 3 spines in *E. taiheizana* Asahina, 1979.

**Figures 16–19.**
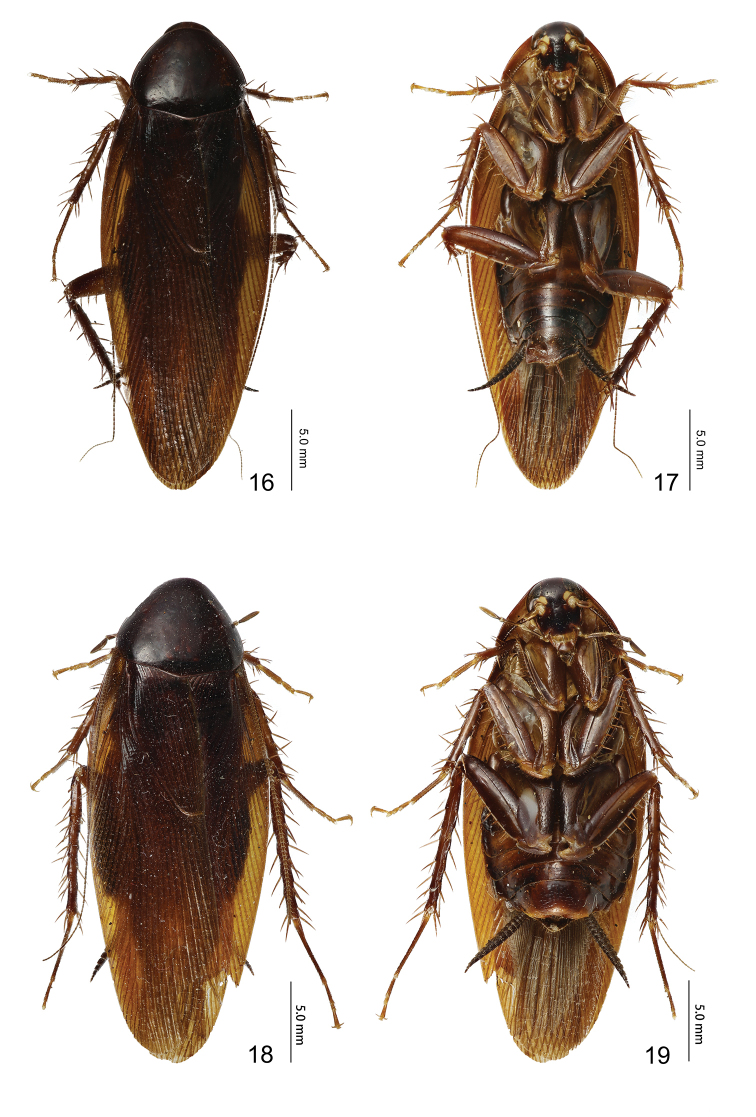
*Episymploce maxima* sp. nov. **16** male, dorsal view **17** male, ventral view **18** female, dorsal view **19** female, ventral view.

#### Description.

Male, pronotum: length × width: 5.2–6.0 × 6.0–6.5 mm; tegmen: 23.5 mm; overall length (including tegmen): 27.8–28.5 mm. Female, pronotum: length × width: 5.5–6.6 × 6.0–6.6 mm; tegmen: 23.4 mm; overall length (including tegmen): 27.8–29.5 mm.

Large size. Body dark brown, head extending beyond the pronotum, vertex tawny, ocellus yellow, face dark brown, interocular space is 3/4 of ocellus space, antenna base dark brown, a pair of symmetrical reddish-brown dots next to the antenna sockets, antenna sockets slightly wider than ocellus width. Pronotum approximate triangular, hind margin wide, dark brown. Tegmina and wings fully developed, tegmina extending beyond the end of abdomen; hind wing with radius vein branched near middle; medial vein simple; cubitus anterior vein with five complete and two incomplete branches, triangular apical area small. Anteroventral margin of front femur Type A3; the first tarsus of the hind leg longer than the sum of the rest tarsi; tarsal claws symmetrical and unspecialized, arolium and pulvillus present. The T1 unspecialized; T7 specialized with numerous hairs in the intermediate region (Fig. 22); right and left lateral plates of the T9 are similar, hind margins truncate, posterior corners rounder (Fig. 23). Male supra-anal plate symmetrical, middle of the hind margin concave (Fig. 24); subgenital plate asymmetrical, two styles on the left side of the hind margin, with some spines in the interstylar margin (Fig. 25); left aedeagus hook-shaped (Fig. 26); median aedeagus exposed, extending beyond the supra-anal plate, spicular (Fig. 27). Female similar to male; supra-anal plate and subgenital plate symmetrical, hind margin round, apex with small concavity (Figs 29, 30).

#### Etymology.

Species name *maxima* refers to its large size, currently the largest species in *Episymploce*.

#### Distribution.

China (Guangxi).

**Figures 20–30.**
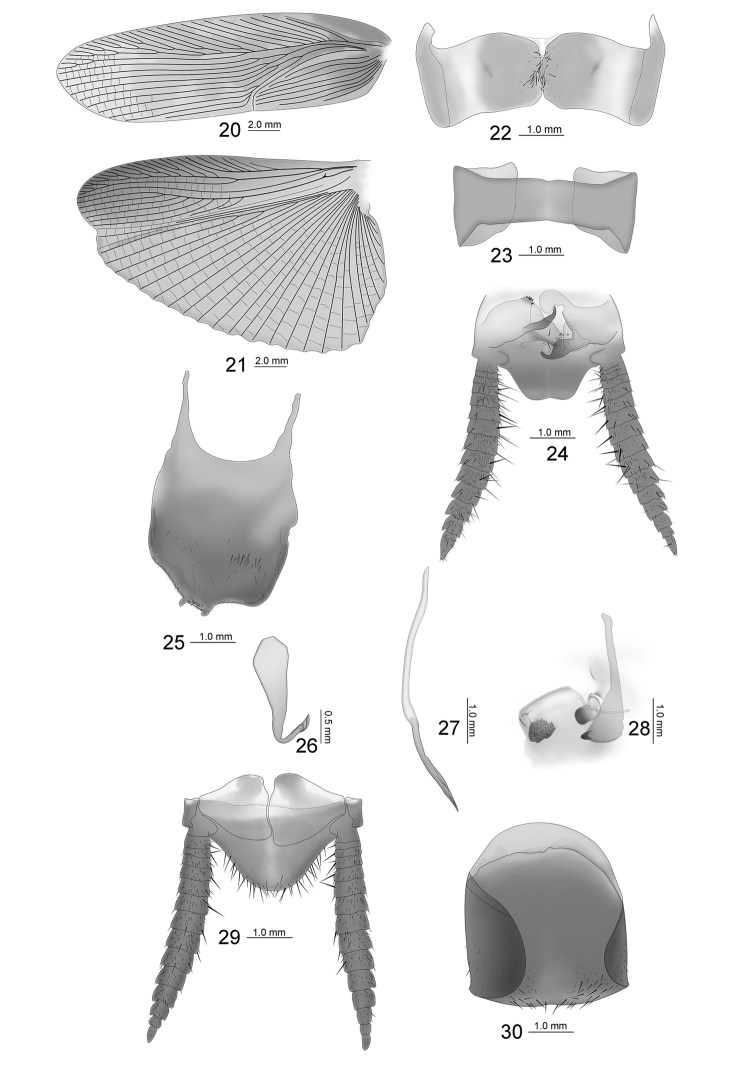
*Episymploce maxima*, sp. nov. **20** tegmen **21** hind wing **22** male, T7, dorsal view **23** male, T9, dorsal view **24** male, supra-anal plate and paraprocts, ventral view **25** male, subgenital plate, dorsal view **26** male, left aedeagus **27** median aedeagus **28** male, right aedeagus **29** female, supra-anal plate and paraprocts, ventral view **30** female, subgenital plate, ventral view.

## Discussion

The genus *Symploce* was established before the genus *Episymploce*, but it turned out that many species from other genera of cockroaches were included (Roth 1984). In 1950, Bey-Bienko (1950) established the genus *Episymploce*, and pointed out the difference between *Episymploce* and *Symploce* in the hind wings, the irregularly branched radial of the tegmina, and the conversion of the hind lateral processes of the T9 into spines. Subsequently, Asahina (1979) redefined *Episymploce* and dissected the male genitalia in detail, providing more reliable features for distinguishing *Symploce* from *Episymploce*. Roth (1986) considered that Bey-Bienko put too much emphasis on wing venation when distinguishing *Episymploce* from *Symploce*. He considered that the wing venation could not be used to distinguish *Episymploce* from *Symploce*, and suggested the symmetry of the supra-anal plate should be considered. He transferred *E. marginata* Bey-Bienko, 1957, *E. popovi* Bey-Bienko, 1957 and *E. ligulata* Bey-Bienko, 1957 to *Symploce*. In 1984 and 1986, Roth (1984, 1986) respectively collated and supplemented the characteristics of the abdominal tergum, wing venation, anteroventral margin of front femur, and supra-anal plate to distinguish *Symploce* from *Episymploce*. More specializations of the male abdominal tergum were observed in *Symploce* than in *Episymploce*. In 1997, Roth rejected that symmetry of the supra-anal plate distinguished between *Episymploce* and *Symploce*, and returned *E. marginata* Bey-Bienko, 1957, *E. popovi* Bey-Bienko, 1957 and *E. ligulata* Bey-Bienko, 1957 to *Episymploce*. We do not think it is appropriate to distinguish one genus from another only by a single feature as there are many similarities between the characteristics of *Episymploce* and *Symploce*.

In 1985, Roth (1985) compared the characteristics of *Blattella*, *Symploce*, *Parasymploce* and *Episymploce*. He considered that the difference between *Parasymploce* and the other three is that the supra-anal plate is symmetrical and the T7 was always specialized. Roth (1995) considered *Aristiger* and *Parasymploce* were synonyms of *Hemithyrsocera*. The supra-anal plate of *E. sichuanensis* sp. nov. is asymmetrical, which is obviously different from *Hemithyrsocera*. *Episymploce maxima* sp. nov. has similar features to *Hemithyrsocera* on the supra-anal plate and T7, but *E. maxima* sp. nov. has 5 complete and 2 incomplete branches in the cubitus anterior vein of the hind wing, while the cubitus anterior vein of the hind wings of *Hemithyrsocera* have no branches or 1–3 complete branches, and no incomplete branches. The supra-anal plate of *E. sichuanensis* sp. nov. is asymmetrical and T1 unspecialized, T7 and T9 are specialized. In the diagnosis of *Symploce* (Roth, 1984), the supra-anal plate was described as symmetrical, rarely asymmetrical, and T7 and T9 without specialization at the same time. *Episymploce maxima* sp. nov. was similar to the genus *Symploce* in regard to the supra-anal plate, but the subgenital plate of *Symploce* has a highly specialized style, while the subgenital plate of *E. maxima* sp. nov. has a simple style and T1 is unspecialized, T7 specialized, and the left and right plate of T9 are similar. We think that these two new species do not agree with the characteristics of *Symploce* and *Hemithyrsocera*, whereas they do agree with the characteristics of the genus *Episymploce*.

## Supplementary Material

XML Treatment for Episymploce

XML Treatment for Episymploce sichuanensis

XML Treatment for Episymploce maxima
